# Weighting of Criteria for Disease Prioritization Using Conjoint Analysis and Based on Health Professional and Student Opinion

**DOI:** 10.1371/journal.pone.0151394

**Published:** 2016-03-11

**Authors:** Nadine Stebler, Gertraud Schuepbach-Regula, Peter Braam, Laura Cristina Falzon

**Affiliations:** 1 Veterinary Public Health Institute, University of Bern, Liebefeld, Switzerland; 2 Federal Food Safety and Veterinary Office, Liebefeld, Switzerland; Univ. Prince Edward Island Atlantic Veterinary College, CANADA

## Abstract

Disease prioritization exercises have been used by several organizations to inform surveillance and control measures. Though most methodologies for disease prioritization are based on expert opinion, it is becoming more common to include different stakeholders in the prioritization exercise. This study was performed to compare the weighting of disease criteria, and the consequent prioritization of zoonoses, by both health professionals and students in Switzerland using a Conjoint Analysis questionnaire. The health professionals comprised public health and food safety experts, cantonal physicians and cantonal veterinarians, while the student group comprised first-year veterinary and agronomy students. Eight criteria were selected for this prioritization based on expert elicitation and literature review. These criteria, described on a 3-tiered scale, were evaluated through a choice-based Conjoint Analysis questionnaire with 25 choice tasks. Questionnaire results were analyzed to obtain importance scores (for each criterion) and mean utility values (for each criterion level), and the latter were then used to rank 16 zoonoses. While the most important criterion for both groups was “Severity of the disease in humans”, the second ranked criteria by the health professionals and students were “Economy” and “Treatment in humans”, respectively. Regarding the criterion “Control and Prevention”, health professionals tended to prioritize a disease when the control and preventive measures were described to be 95% effective, while students prioritized a disease if there were almost no control and preventive measures available. Bovine Spongiform Encephalopathy was the top-ranked disease by both groups. Health professionals and students agreed on the weighting of certain criteria such as “Severity” and “Treatment of disease in humans”, but disagreed on others such as “Economy” or “Control and Prevention”. Nonetheless, the overall disease ranking lists were similar, and these may be taken into consideration when making future decisions regarding resource allocation for disease control and prevention in Switzerland.

## Introduction

Disease prioritization exercises have been used by several organizations and national research groups to inform surveillance and control measures, while optimizing resource allocation [1–3]. These priority setting exercises are a multi-dimensional task, as they need to take into consideration several factors which may sometimes be difficult to compare. These include: clinical factors, such as severity of the disease; epidemiological factors, such as incidence of the disease; and economic parameters [2, 4].

Several disease prioritization methods have been described, including workshops [2], Delphi panels [5], and questionnaires [6, 7]. While most of the described methodologies are based on expert opinion [2, 6–11], several groups have recognized that different stakeholders may perceive risks differently, which could lead to different priorities [12, 13]. This is of particular relevance when considering the perception of zoonotic diseases, as these have a large impact on numerous life sectors, including health and economy. Moreover, human behavior may largely affect the spread, prevention, and control of these diseases [13]. Consequently, future refinement of priority setting techniques for zoonoses should incorporate values of multiple stakeholders within their assessment [14], particularly of those who are directly affected, such as veterinarians and farmers [3].

A few working groups have already included multiple stakeholder opinions within their zoonotic disease prioritization exercises. In the Netherlands, Havelaar et al. [15] included both experts and medicine and veterinary medicine students in their quantitative priority setting method. More recently, Ng and Sargeant [2, 16, 17] included experts and the general public in qualitative focus groups to elicit information on which criteria were relevant for disease prioritization, and these were then followed by an online Conjoint Analysis (CA) questionnaire to weight these criteria.

Conjoint Analysis was developed in the sixties by the mathematical psychologists Luce and Tukey [18], and is often used in the field of marketing and consumer research to obtain information on people’s preferences regarding a certain type of product. Each product is described by a series of attributes, such as price, size or color, and stakeholders are then asked to choose between products possessing different levels of the same attributes. By choosing one product over another, people inadvertently provide information on which attributes they consider more important [19], and this information can then be used for marketing purposes, but also prioritization exercises.

In Switzerland, the Swiss Food Safety and Veterinary Office and the cantonal veterinary offices described the need to prioritize zoonotic disease in the recently published document “Animal Health Strategy 2010+” [20]. As the prioritization should reflect the opinion of Swiss policy makers and other Swiss stakeholders, thus incorporating the local situation, results published by other countries could not be extrapolated to Switzerland. Priorities in Switzerland might differ from other countries because herd sizes are smaller compared with other European countries [21], and because there are many animal movements due to alpine pasturing [22, 23]. Moreover, Switzerland is declared as officially free from diseases such as bovine Tuberculosis or Glanders, though the risk of introduction still persists due to the intensive international trade and tourism. It is therefore important to take these differences into consideration when prioritizing diseases for control and surveillance in Switzerland.

In a first step to address this request for prioritization of zoonoses, a literature search and modified Delphi panel based on expert opinion were performed to identify and evaluate disease criteria relevant for disease prioritization [24]. In a second step, the aims of this study were to: (i) weight these disease criteria using a CA questionnaire based on both Swiss health professional and student opinion, and (ii) use these weighting scores to rank 16 notifiable or emerging zoonotic diseases in Switzerland. The rationale for this study was to provide Swiss veterinary public health policy makers with data to update the current list of notifiable and emerging zoonotic diseases, based on the opinion of multiple Swiss stakeholders.

## Materials and Methods

### Selection of the criteria used in the prioritization process and questionnaire development

**Fig 1:**
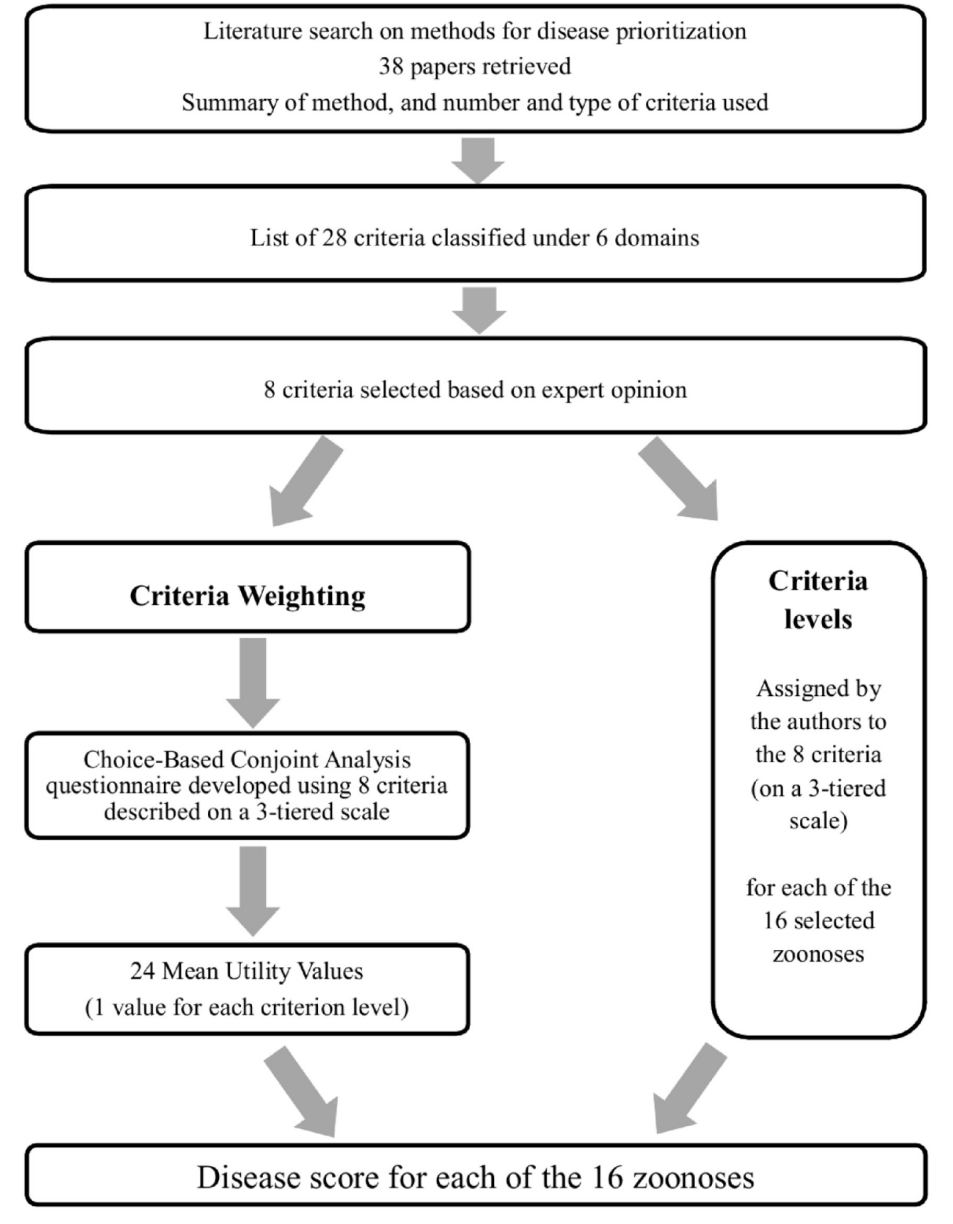
A schematic diagram to illustrate the weighting and prioritization process used for prioritization of zoonotic diseases based on health professional and student opinion in Switzerland. The final disease score for each zoonosis was obtained by matching the levels for the different criteria with the respective mean utility values obtained from a choice-based Conjoint-Analysis questionnaire.

The methods for criteria selection have been described by Stebler et al. [24], and are summarized schematically in Fig 1. Briefly, 28 criteria relevant for disease prioritization and classified under 6 domains (“Burden of disease in humans”, “Burden of disease in animals”, “Epidemiology”, “Control and prevention”, “Economy” and “Society”), were identified following a thorough literature search. These criteria were then evaluated by experts within a modified Delphi panel to ensure that the compiled list was adequate and complete, and this was followed by a weighting exercise.

A questionnaire based on the CA methodology was developed to obtain weighting scores for each criterion from both health professionals and students. Since an important assumption of this method is that all assessed criteria must be independent to avoid collinearity, only 8 of the original 28 criteria were included in the questionnaire. The final eight criteria included in the questionnaire were: “Severity of the disease in humans”, “Economy”, “Treatment in humans”, “Incidence of the disease in humans”, “Control and prevention”, “Severity of the disease in animals”, “Incidence of the disease in animals” and “Transmission”. For each of the eight criteria, and based on input from a social science expert, a three-tiered measurement scale was developed.

**Fig 2:**
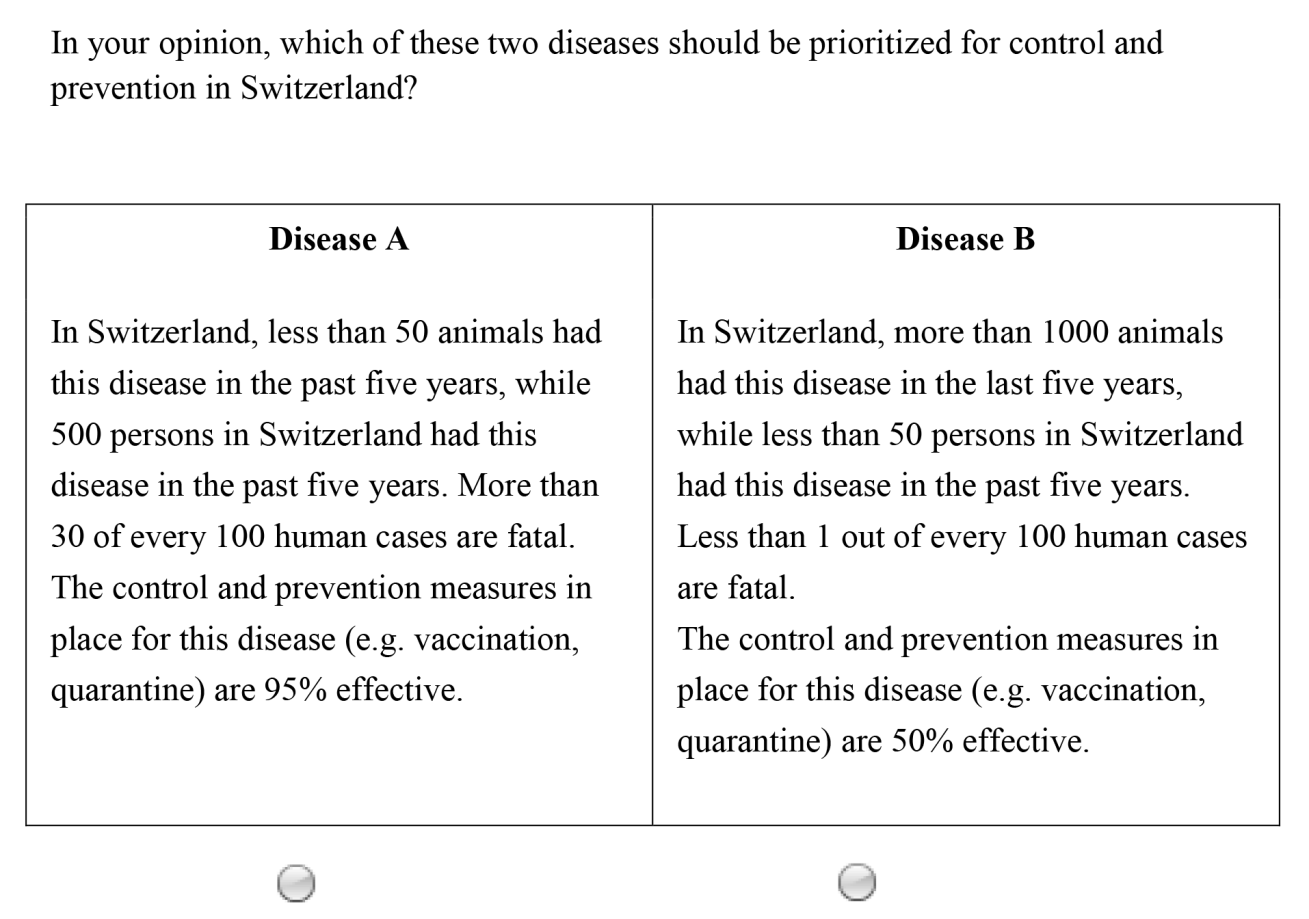
An example of a choice task used in the Choice-Based Conjoint Analysis questionnaire to obtain expert and student opinion on zoonotic disease prioritization in Switzerland. In each choice task, two fictitious diseases were presented (Disease A and Disease B), each of which was described using different levels of four out of eight criteria; the disease criteria and levels varied in each choice task. Note: The questionnaire used in this study was in German, and this choice task has been translated into English specifically for publication.

Due to the fairly large number of criteria to be assessed, a partial-profile Choice-Based Conjoint Analysis (CBC) survey was developed using Sawtooth Software CBC version 8.2.4. The partial-profile survey allows one to only assess part of the criteria in each choice task, while ensuring that all criteria are equally represented. The questionnaire contained 25 choice tasks, each comparing two fictitious diseases (Disease A and Disease B) with a description of four out of the eight criteria. Participants were asked to select the disease which they considered had the higher priority for surveillance and control (see Fig 2 for an example). The disease criteria and levels assessed varied in each choice task.

The questionnaire was pre-tested to assess the number and clarity of choice tasks, and suggestions were incorporated into the final version. Two versions of the questionnaire, with a randomized order of choice tasks in each, were created using Sawtooth Software CBC version 8.2.4, and this was done to reduce systematic bias since first presented attributes tend to be given more attention. These questionnaires were distributed as a paper-and-pencil survey for both logistical and practical reasons, and all questionnaires were in German. The strength of design (D-efficiency) calculated for 170 respondents was 684.70669 relative to a full-orthogonal design, with a standard error <0.05 for each criterion level. The questionnaire is available from the corresponding author upon request.

### Survey population

The stakeholders represented in this study included health professionals and students; the latter were considered representative of laymen with an interest in agriculture and/or veterinary medicine, but with no prior expertise in zoonotic diseases.

For the health professional group, the questionnaire was administered in person to the six experts from the Swiss Food Safety and Veterinary Office who participated in the previously held modified Delphi panel [24], and to experts from the Federal Office of Public Health. Criteria used in the selection of these experts included: (i) expertise in the sectors of: animal health, monitoring of epizootics and zoonoses, food safety, knowledge translation and transfer, and communication, (ii) availability, and (iii) willingness to participate. The questionnaire was also sent by mail to all German-speaking and bilingual (German- and French-speaking) Swiss cantonal official veterinarians and official physicians, who are responsible for the cantonal surveillance of animal and human health, respectively. Participants had to be German-speaking since the questionnaire was only available in German, and this was done to avoid possible translation bias.

The students were represented by first year veterinary students from the Vetsuisse Faculty (at both the University of Bern and University of Zurich), as well as first-year agronomy students from the School of Agricultural, Forest and Food Sciences in Bern. A presentation explaining the purpose of the research project and questionnaire preceded the distribution of the questionnaire. All questionnaires were kept anonymous, and a 10 Swiss Francs (8€) voucher was offered as an incentive to all the students who completed the questionnaire.

According to Swiss Legislation, no ethical clearance was required for this study since it did not involve collection of sensitive data. All participants were involved voluntarily and gave consent for their responses to be used for research purposes and to be published.

### Statistical analyses

Data obtained from each questionnaire were entered into an Excel spreadsheet (Microsoft Office Excel, 2007), saved as a csv-file, and then imported into Sawtooth Software CBC/HB version 8.2.4. This software uses a Hierarchical Bayes model to estimate the part-worth utility values (ß) and importance scores of each respondent, based on which of the two diseases described in each choice task was selected for prioritization, and the corresponding attributes and levels used to describe that disease.

The Hierarchical Bayes model has an upper- and lower-level model; the former models the variation in preference between respondents (between variation) and serves as a prior information, while the latter models the variation between questions answered by the same respondent (within variation), and provides a likelihood. The model then determines posterior probability values based on the most optimal weight of the upper- and lower-level models, and these are equivalent to the Mean Utility Values (MUVs). These final individual-level parameter estimates represent the relative influence each criterion level had on respondent choices, with higher values indicating a stronger influence on choice.

Importance scores are then estimated for each criterion by dividing the difference between the highest and lowest criterion level mean utility value, by the sum of all mean utility value ranges for all criteria. Therefore, the greater the magnitude between the higher and lower MUVs, the greater the importance score, indicating that these criteria had a stronger influence on which disease was prioritized.

To assess for potential confounding, the effect of a few covariates on the MUVs obtained from the student group was evaluated. The covariates assessed included: subject (veterinary medicine vs. agronomy), farming background (yes vs. no), gender and age. This was not possible for the health professional group since the sample size was too small. The goodness-of-fit of the model was based on the expected percent certainty and Root LikeliHood (RLH). The expected percent certainty is 0% for a chance model, and 100% for a perfect model, while the expected RLH is 0.5 for a chance model (1 divided by the number of choice tasks, which in this study was 2), and 1.0 for a perfect model.

### Selection and scoring of zoonoses

Sixteen zoonoses, representing either one of the four categories of notifiable diseases or the category of emerging diseases in Switzerland [25], were selected for evaluation in this study. These diseases were selected either because of their current status in Switzerland and in neighboring countries, or due to their relative importance in other recently published ranking lists [3, 15, 26].

Each of these 16 zoonoses was evaluated using the same eight 3-tiered criteria developed for the CA questionnaire. Scoring was done independently by two of the authors (NS and LCF), and decisions were based on information found in recently published articles on the topic [3, 27], official web-sites [28–30], disease reports [31] and a textbook [32]. The levels assigned to each criterion, for each disease, were compared during a consensus process between the two authors, and any disagreement was resolved through discussion and by consultation with a third co-author (GS-R) (The levels assigned to the 8 criteria for each of the 16 zoonotic diseases are available in S1 Table).

### Ranking of zoonoses for disease prioritization

Two zoonotic disease ranking lists were created using the MUVs obtained from the CBC questionnaires administered to the health professional and student groups, respectively.

Specifically, a final disease score was created by taking the MUV for the level that matched the score assigned to the eight criteria, for each disease and criterion, respectively. The 8 MUVs were added up to create a final disease score, which was then used to rank the 16 zoonotic diseases. For sensitivity analysis purposes, the 95% lower and upper confidence intervals were applied to the MUVs, and any changes in the ranking lists were noted.

## Results

### Survey population

Thirty two health professionals participated in this study. These included: five public health experts from the Federal Office of Public Health and seven veterinary experts from the Swiss Food Safety and Veterinary Office. Additionally, the questionnaire was completed and returned by 6 out of 19 (32%) German-speaking cantonal official physicians, and 14 out of 15 (93%) German-speaking cantonal veterinary officers. Of these 32 experts, 21 (66%) were male and 11 (34%) were female. The mean age of this group was 52.2 years (range = 35 to 65 years).

A total of 204 students were present in class when the questionnaire was administered, and all completed the questionnaire. Of these, 136 (67%) were first-year veterinary students (of which 60 and 76 students were from the University of Bern and the University of Zurich, respectively), and 68 (33%) were first-year agronomy students. Of the 204 students, 49 (24%) were male and 155 (76%) were female. The mean age was 21.4 years (range = 18 to 37 years).

### Disease criteria Importance Scores and Mean Utility Values based on Health Professional opinion

The three highest-weighted criteria by health professionals were “Severity of the disease in humans” (importance score = 16.52), followed by “Economy” (importance score = 16.41), and “Treatment in humans” (importance score = 14.66), while the criterion “Transmission” was the least influential (importance score = 8.43) (Table 1).

**Table 1:** The eight criteria, and the three levels used to describe each one of them, included in a Choice-Based Conjoint Analysis questionnaire on zoonotic disease prioritization in Switzerland, and their Rank (based on the Importance Score), the Importance Score (and Standard Deviation), and the Mean Utility Values of each criterion level, assigned by the health professional and student groups, respectively.

	Health Professionals			Students		
Criteria (and the levels used to describe them)	Rank	Importance Score (Standard Deviation)	Mean Utility Value	Rank	Importance Score (Standard Deviation)	Mean Utility Value
**Severity of the disease in humans**	1	16.52 (6.39)		1	17.95 (5.37)	
Fatality in humans <1%			-71.51			-78.21
Fatality in humans = 20%			14.86			13.69
Fatality in humans >30%			56.66			64.52
**Economy**	2	16.41 (5.25)		8	7.80 (3.78)	
No impact on trade			-67.59			-28.46
Slight restrictions			5.65			2.08
Stand-still			61.93			26.39
**Treatment in humans**	3	14.66 (4.96)		2	15.15 (5.64)	
Lasts less than 1 week			-41.15			-45.78
Lasts for 2 weeks			-26.66			-23.76
Lasts more than 4 weeks			67.80			69.54
**Incidence of the disease in humans**	4	13.00 (3.87)		5	12.50 (3.58)	
Incidence in humans in the last 5 years in Switzerland <50 persons			-56.54			-57.29
Incidence in humans in the last 5 years in Switzerland = 500 persons			22.45			17.66
Incidence in humans in the last 5 years in Switzerland >1000 persons			34.10			39.63
**Control and prevention**	5	11.78 (5.26)		6	10.41 (5.66)	
Measures are 95% effective			32.75			-14.43
Measures are 50% effective			9.01			2.06
Measures are 5% effective			-41.76			12.37
**Severity of the disease in animals**	6	10.53 (4.39)		4	13.00 (4.40)	
Fatality in animals <1%			-44.70			-54.96
Fatality in animals = 20%			17.42			8.51
Fatality in animals >30%			27.28			46.45
**Incidence of the disease in animals**	7	8.67 (5.34)		3	13.67 (5.96)	
Incidence in animals in the last 5 years in Switzerland <50 animals			-32.44			-55.62
Incidence in animals in the last 5 years in Switzerland = 500 animals			13.22			5.25
Incidence in animals in the last 5 years in Switzerland >1000 animals			19.22			50.36
**Transmission**	8	8.43 (4.05)		7	9.53 (4.71)	
By direct contact			-17.61			-23.14
By indirect contact			-10.78			-6.63
Air-borne			28.39			29.78

Twenty-four MUVs were estimated (3 levels for each of the 8 criteria), and these values ranged from -71.51 to 67.80 (Table 1). Seven of the eight criteria were more likely to be selected when described using the third level, compared to when they were described with the first or second level. Taking “Treatment in humans” as an example, a disease was selected by more health professionals when it was described using the third level of this criterion (i.e. “Treatment lasts for more than four weeks”; MUV = 67.80), compared to when it was described using the first (i.e. “Treatment lasts less than a week”; MUV = -41.15) or second level (i.e. “Treatment lasts two weeks”; MUV = -26.66). The only exception was the criterion “Control and prevention”, where a disease was more likely to be selected by the health professionals if it was described using the first level of this criterion (i.e. “Measures are 95% effective”; MUV = 32.75), as opposed to when it was described using the second (i.e. “Measures are 50% effective”; MUV = 9.01), or third level (i.e. “Measures are 5% effective; MUV = –41.76).

The overall fit of the model using health professional data was above satisfactory, with a percent certainty fit of 83.6% and an RLH of 0.85.

### Disease criteria Importance Scores and Mean Utility Values based on Student opinion

The three criteria that were weighted highest by the student group were “Severity of the disease in humans” (importance score = 17.95), followed by “Treatment in humans” (importance score = 15.15), and “Incidence of the disease in animals” (importance score = 13.67), while “Economy” was the least influential criterion (importance score = 7.80) (Table 1).

The MUVs for the 24 levels ranged from -78.21 to 69.54 (Table 1). For all criteria, the third level always had the highest MUV, indicating that priority was given to those diseases that were described using the third level. Unlike the health professionals, this was also the case for the criterion “Control and prevention”, where students were more likely to prioritize a disease if it was described using the third level (i.e. “Measures are 5% effective”; MUV = 12.37), rather than with the first (i.e. “Measures are 95% effective”; MUV = –14.43), or second level (i.e. “Measures are 50% effective”; MUV = 2.06).

None of the covariates assessed in the CA was associated with how the students’ prioritized the disease criteria. For the student group, the model fit was also above satisfactory, with a percent certainty fit of 79.9% and an RLH of 0.8.

### Scoring and ranking of the zoonotic diseases

The ranking lists of zoonotic diseases based on the MUVs from the health professional and student group, and the respective difference in rank, are presented in Table 2. For both groups, the two most important diseases were Bovine Spongiform Encephalopathy (BSE) and Rabies, while Bovine Tuberculosis and Glanders were ranked third for the health professionals and students, respectively. Newcastle Disease, West Nile Fever and Avian Chlamydiosis were ranked as the bottom three in both lists. Overall, 6 of the 16 diseases had the same rank in both groups, while the other 10 diseases were ranked within 3 positions of each other. When the upper and lower 95% confidence intervals were applied to the MUVs based on the health professionals’ opinion, 12 of the 16 diseases did not change their position in the ranking list, 3 diseases moved one position, and only 1 disease (Toxoplasma) was ranked 4 positions lower with the upper confidence limit. When the 95% confidence intervals were applied to the MUVs based on the students’ opinion, 12 of the 16 diseases did not change their position in the ranking list, while 4 diseases moved 1 position (results not shown).

**Table 2:** The 16 notifiable or emerging zoonoses which were ranked using the sum of the Mean Utility Values obtained from a Choice-Based Conjoint Analysis questionnaire administered to both health professionals and students, and their relative rank difference, in a study on prioritization of zoonoses in Switzerland.

Rank	Ranking based on Mean Utility Values from Health Professionals	Final Disease Score	Ranking based on Mean Utility Values from Students	Final Disease Score	Difference in Rank (relative to Health Professionals)
1	Bovine Spongiform Encephalopathy	146.66	Bovine Spongiform Encephalopathy	72.93	0
2	Rabies	83.55	Rabies	32.11	0
3	Bovine Tuberculosis	48.58	Glanders	14.28	1
4	Glanders	24.84	Echinococcosis	6.67	2
5	Listeriosis	14.98	Bovine Tuberculosis	-2.21	-2
6	Echinococcosis	-12.6	Avian Influenza	-7.99	1
7	Avian Influenza	-24.94	Listeriosis	-23.66	-2
8	Nipah Virus Encephalitis	-41.63	Q Fever	-50.22	2
9	Toxoplasmosis	-56.1	Nipah Virus Encephalitis	-52.51	-1
10	Q Fever	-67.89	Campylobacteriosis	-99.97	3
11	Leptospirosis	-120.38	Leptospirosis	-117.67	0
12	Salmonellosis	-155.66	Toxoplasmosis	-150.1	-3
13	Campylobacteriosis	-158.91	Salmonellosis	-183.59	-1
14	Newcastle Disease	-204.55	Newcastle Disease	-187.07	0
15	West Nile Fever	-231.11	West Nile Fever	-220.57	0
16	Avian Chlamydiosis	-249.29	Avian Chlamydiosis	-310.86	0

## Discussion

“Severity of the disease in humans” was considered the most important criterion when prioritizing diseases, followed by the criterion “Treatment in humans” (second important criterion for students, and third for health professionals). The criterion “Severity of the disease in humans” is also highly weighted in several other studies [5, 15–17, 33], and this could be since it is a criterion which most people can relate to, regardless of their background or expertise. On the other hand, the weighting of the criterion “Treatment in humans” varies considerably in different studies: it is rated highly in a study from Belgium [33], in the midrange in a study from Canada and the USA [3], and as of negligible importance in a study from Colombia [5], though it should be noted that the way this criteria is categorized in the other studies differs slightly from the categorization used in this study. The perceived importance of treatment might vary between countries as a consequence of differences in health care systems and accessibility to treatments, or due to differences in societal organizations and institutions as a whole.

Health professionals weighted “Economy” as the second most important criterion (importance score = 16.41), while for the students it was the least influential criterion (importance score = 7.80). This difference between the two groups is not surprising, as research has shown that health professionals often take an objectivist approach to risk management, using quantifiable concepts such as costs to assess and measure risk [34]. Therefore, economic components related to disease control would play a more important part in an experts’ decision on whether to prioritize a disease or not, compared to other stakeholders involved.

On the other hand, students considered “Incidence of the disease in animals” and “Severity of the disease in animals” more important, compared to health professionals, as these criteria were weighted third and fourth, respectively. This offers some insight into how future veterinarians and farmers, both important stakeholders in decisions regarding zoonoses prioritization, may perceive disease control and management strategies. Research has shown that, while some control strategies such as culling may be more economically feasible, their implementation has sometimes failed because farmers might prefer more expensive strategies that safeguard their animals’ wellbeing, such as vaccination [35]. Therefore, control strategies that do not take into consideration the difference in priorities given by different stakeholders, and the importance of proper risk communication, might have less support from those involved [35].

Interestingly, health professionals in this study tended to prioritize a disease when the control and preventive measures were described to be 95% effective, while the students prioritized a disease if there were almost no control and preventive measures available. This further highlights how multiple stakeholders may perceive risks differently. Experts often take a more managerial stance, also taking feasibility of disease control into account. On the other hand, lay people tend to have a more subjectivist perception of disease risk, focusing on those risks that are unknown or not controllable. It is therefore important that health professionals engage in a two-way communication with, and understand the concerns of other stakeholders involved, to overcome the possible barriers created by these different risk perceptions [36]. Results from risk prioritization exercises such as this can facilitate risk communication by providing an insight into how to address concerns and priorities of different stakeholders. Lastly, all participants tended to prioritize a disease if it was transmitted via indirect or airborne routes, versus direct routes. This may be because such transmission is perceived to be faster and harder to avoid, compared to transmission which requires direct contact with an infected animal or person.

Two ranking lists were created based on the MUVs from the health professionals and student group, respectively. The two top-ranked diseases were BSE and Rabies, while Bovine Tuberculosis and Glanders were ranked third by the health professionals and students group, respectively (Table 2).

In this study, BSE was ranked first by both groups, and this is because it was assigned the highest level for the criteria “Severity of the disease in humans”, “Severity of the disease in animals”, “Economy” and “Treatment in humans”, particularly since the disease is fatal and no treatment is currently available. Bovine Spongiform Encephalopathy has a high impact on the economy, and has resulted in high public awareness. This was particularly the case in Switzerland since, after the United Kingdom, it was the first country that acknowledged and dealt with cases during the disease epizootic in the nineties, and this led to a complete re-structuring of the Swiss veterinary services. Bovine Spongiform Encephalopathy variant Creutzfeld-Jakob Disease or Transmissible Spongiform Encephalopathies in general, are also among the top ten diseases in studies by Horby et al. [37], Havelaar et al. [15], and Ng and Sargeant [16, 17].

Rabies also received a high weighting in this study because of the high severity of the disease in both humans and animals. Despite this high fatality, rabies is also an easily preventable disease with effective post-exposure prophylaxis treatment in humans and control options available in both dog and wildlife populations [38]. Given the high burden of rabies globally [38], accompanied by the high feasibility to eliminate the disease [39, 40], it is not surprising that rabies would be one of the top prioritized diseases.

While these results were not unexpected for some of the reasons mentioned above, they do highlight that there may be a major difference between the perception of which diseases are important and the actual reality on the ground. In the latest report for zoonoses monitoring in Switzerland [31], the most commonly identified zoonotic diseases were Campylobacteriosis and Salmonellosis, with 7481 and 1271 confirmed human cases respectively. On the other hand, no human cases of BSE variant Creutzfeld-Jakob Disease or Rabies, the two top-ranked diseases in this study, were reported for the same year.

In general, these ranking lists cannot be compared with the results of other disease prioritization studies, as these are often based on different epidemiological characteristics or methodologies. Regarding the latter, there is still no accepted gold standard method for prioritization exercises. Several prioritization exercises often start with a qualitative method, which allows for the collection and refinement of different opinions on the relevance and weighting of disease criteria [10, 27, 41]. Qualitative methods also allow for the assessment of a larger number of criteria, which in turn provides a more accurate assessment of the diseases. However, these methods can be laborious and time-consuming, and may be limited by the number of experts available or willing to participate. Therefore, quantitative methods can serve as an important adjunct to further refine the weighting estimates, and may be easier to implement once the necessary software is available. Moreover, the use of fewer criteria for disease prioritization allows for a quick disease assessment, making the exercise more practical and accessible. Both qualitative and quantitative methods may have internal validity if properly executed; however, external validity might be more easily achieved with a quantitative method as this can be administered to a larger study population. Lastly, results from a quantitative method may sometimes be more easily communicated, compared to qualitative results, as they often evaluate fewer criteria and generate estimates with uncertainty values around them. In our study, the combination of a qualitative study (to identify and evaluate criteria) followed by a quantitative study (to further refine the criteria weighting), proved to be useful for the aim of the Swiss Federal Food Safety and Veterinary Office to use this ranking for decisions regarding allocation of resources to future surveillance and control of zoonoses. For this purpose, it is extremely important that the ranking reflects the opinion of the most important stakeholders, such as experts and veterinary and medical professionals. This was achieved by involving experts in the selection of the criteria (the qualitative step), and by using relevant stakeholder groups for the weighting of the criteria (the quantitative step). Stakeholder involvement could be further improved by also including a representative group of consumers in the prioritization exercise.

In this study, the CA questionnaire was developed using eight criteria described on a three-tiered scale. This was done to reduce the number of choice tasks within each questionnaire and to ensure that all criteria assessed in the CA questionnaire were independent. Despite this, we recognize that some of the criteria such as “Severity of the disease”, “Transmission” and “Economy” might still not be completely independent (e.g. a more severe disease is likely to have a greater impact on economy, compared to a less severe disease).

One of this study’s limitations was the relatively small sample size of the health professional group, compared to the student group. Nonetheless, the statistical analysis of a CA using Hierarchical Bayes models can still be effective for small sample sizes [19], and this was demonstrated by the overall fit of the model which was above satisfactory for both groups. Another limiting factor was the low response rate of cantonal physicians (31%), compared to that of the cantonal veterinarians (93%). This may be explained by the fact that the veterinarians are more aware of the importance of zoonoses, and were therefore more willing to participate in the study. We recognize that this discrepancy between animal and human health professionals may have led to non-response bias, as veterinarians were more likely to prioritize “Economy”, compared to human health professionals. However, given the small sample size, we were unable to perform a stratified analysis for the animal and human health professionals. We also recognize that the omission of practicing veterinarians and farmers from this study might have led to selection bias due to choice of comparison group, while limiting the external validity of this study, but their inclusion was not possible due to logistical and financial constraints of this study. Misclassification bias due to leading questions in the questionnaire might also have been possible. However, the latter was dealt with by using the narrative form to describe the fictitious diseases in each choice task, and by randomizing the order of the choice tasks in each questionnaire.

Overall, this study provided information on which criteria are relevant for disease prioritization, and their respective weighting based on both health professional and student opinion. The study results indicate that while some criteria were weighted similarly by both groups, the weighting of other disease criteria varied considerably. Moreover, the perception of “Control and Prevention” measures was dissimilar between the two groups. Nonetheless, the ranking of diseases was similar between the two groups, especially for the top two diseases. These findings will inform future policy-making on the prioritization of zoonotic disease control and surveillance in Switzerland, thus helping to optimize future allocation of resources. Moreover, the results of this study have contributed to discussions regarding which criteria should be used to establish a list of “Diseases of Union Concern”, as part of the new European Animal Health Law.

## Supporting Information

S1 Table Scoring of zoonotic diseases. This table presents the levels (from 1–3) assigned to the 8 criteria on disease prioritization for each of the 16 zoonotic diseases included in the study on zoonotic prioritization in Switzerland.
(DOCX)
