# Supplementary material for: Weighting of Criteria for Disease Prioritization Using Conjoint Analysis and Based on Health Professional and Student Opinion
Source: PLoS One. 2016 Mar 11;11(3):e0151394. doi: 10.1371/journal.pone.0151394 (PMC4788351; doi:10.1371/journal.pone.0151394)
Supplement: S1 Table — This table presents the levels (from 1–3) assigned to the 8 criteria on disease prioritization for each of the 16 zoonotic diseases included in the study on zoonotic prioritization in Switzerland. (DOCX) [file pone.0151394.s001.docx]

|  | **1** | **2** | **3** |
| --- | --- | --- | --- |
| **Severity of the disease in humans** | 0.99*symptomatic with flu-like symptoms, respiratory distress and fever, chills, headache and weakness; 0.01*cardiovascular disorders, hepatitis, and 1% with lethal outcome |  |  |
| **Economy** |  | Restrictions at farm level (OIE and EU regulations on specific trade and import conditions) |  |
| **Treatment in humans** | 0.99*total recovery without relapse; 0.01*difficult treatment (supportive therapy and antibiotics) |  |  |
| **Incidence of the disease in humans** | Sporadic incidence with <1 cases/100,000 inhabitants |  |  |
| **Control and prevention** |  | Moderate in humans where prevention is based on education and proper hygiene; low in animals as there is no effective vaccine and prevention is based on EU/OIE regulations regarding import of birds, quarantine, etc.; certain regulations in place for Newcastle disease have spillover effects for the control of Chlamydia. |  |
| **Severity of the disease in animals** | 0.8*asymptomatic; 0.2*severe illness (respiratory or GI symptoms) |  |  |
| **Incidence of the disease in animals** | In the last 15 years, there were 2-10 cases/year; diminishing incidence trend for the last 20 years |  |  |
| **Transmission** | Human infections most commonly acquired via direct route from inhalation of infected avian excreta or fomites. |  |  |

**Avian Chlamydiosis**

# S1 Table. Scoring of zoonotic diseases. This table presents the levels (from 1-3) assigned to the 8 criteria on disease prioritization for each of the 16 zoonotic diseases included in the study on zoonotic prioritization in Switzerland.

**Bovine Spongiform Encephalopathy**

|  | **1** | **2** | **3** |
| --- | --- | --- | --- |
| **Severity of the disease in humans** |  |  | Fatal or severe long term damages |
| **Economy** |  |  | Possible nationwide ban/standstill, loss of official disease status |
| **Treatment in humans** |  |  | No treatment |
| **Incidence of the disease in humans** | Sporadic incidence; <1/100,000 cases |  |  |
| **Control and prevention** | Effective prevention tools through active surveillance and ban on meat protein meal in animals |  |  |
| **Severity of the disease in animals** |  |  | Fatal |
| **Incidence of the disease in animals** | Sporadic incidence; <1/100,000 cases |  |  |
| **Transmission** |  | Food-borne (indirect) transmission |  |

**Campylobacteriosis (*C. jejuni*; *C. coli*)**

|  | **1** | **2** | **3** |
| --- | --- | --- | --- |
| **Severity of the disease in humans** | 0.8*symptomatic, very mild course of disease, health care utilization is rare; 0.2*symptomatic and therapy is recommended |  |  |
| **Economy** | Restrictions only at animal level, no hindrance to movement of animals and animal products |  |  |
| **Treatment in humans** |  | Often effective treatment with total recovery and without relapse, but complications may occur in certain risk groups |  |
| **Incidence of the disease in humans** |  |  | Epidemic, incidence=90/100,000 (with 7481 cases reported in 2013) |
| **Control and prevention** |  | Prevention in humans possible through various control measures during processing; no vaccine for animals |  |
| **Severity of the disease in animals** | 0.9*asymptomatic or very mild course of disease; 0.1*mild clinical disease but good prognosis |  |  |
| **Incidence of the disease in animals** |  |  | Prevalence 10-20% (can reach 100% in pigs) with stable incidence rates in the last 5 years |
| **Transmission** |  | Transmission by direct or indirect contact; food-borne |  |

**Echinococcosis**

|  | **1** | **2** | **3** |
| --- | --- | --- | --- |
| **Severity of the disease in humans** |  | Severe illness, hospitalization is necessary and fatal if complications arise |  |
| **Economy** | Restrictions only at animal level |  |  |
| **Treatment in humans** |  |  | Long-term anthelmintic treatment or surgery but not always 100% effective |
| **Incidence of the disease in humans** | Sporadic; since 2000 there have been 10-20 new cases/year |  |  |
| **Control and prevention** |  | Prevention in humans by washing fruit and vegetables and implementation of sanitary measures; dogs can be treated with praziquantel; sheep carcass control at slaughter plants though important role of wildlife in transmission makes it hard to control in certain areas |  |
| **Severity of the disease in animals** |  | 0.5*asymptomatic (in end hosts); 0.5*severe illness and poor prognosis (in intermediate hosts) |  |
| **Incidence of the disease in animals** |  | Prevalence <10% with a slight increase in trend; 11cases reported in 2013, but higher prevalence in fox population |  |
| **Transmission** |  | Direct transmission or food-borne |  |

**Glanders**

|  | **1** | **2** | **3** |
| --- | --- | --- | --- |
| **Severity of the disease in humans** |  | Severe illness (with high fever, ulceration, swelling of the lymph nodes); may be fatal if treatment unavailable or resistant to antibiotics |  |
| **Economy** |  | Restrictions at the herd level |  |
| **Treatment in humans** |  |  | Long therapy (of approx. 12-20 weeks); limited efficacy (case-fatality with treatment is 30-40%) |
| **Incidence of the disease in humans** | Disease is not present in Switzerland and in adjacent territories |  |  |
| **Control and prevention** |  | No vaccine is available in humans but disease can be prevented using protective clothing and following established guidelines; in animals no vaccine is available either but special movement measures and effective bans may be implemented to monitor any animals entering the country |  |
| **Severity of the disease in animals** |  |  | Severe illness with three forms (nasal, pulmonary and cutaneous); high fatality rate especially if untreated |
| **Incidence of the disease in animals** | Disease is not present in Switzerland |  |  |
| **Transmission** |  | Transmission by direct contact with infected animals and indirectly via communal food and water sources |  |

**Avian Influenza**

|  | **1** | **2** | **3** |
| --- | --- | --- | --- |
| **Severity of the disease in humans** |  | 0.6*symptomatic (fever and respiratory signs); 0.4*severe illness (pneumonia) |  |
| **Economy** |  |  | Zone standstill, loss of official status |
| **Treatment in humans** |  | 0.6*effective treatment with total recovery; 0.4*long therapy with antivirals or hyper-immune plasma |  |
| **Incidence of the disease in humans** | Not present in Switzerland |  |  |
| **Control and prevention** |  |  | Vaccines are sometimes produced for humans but only in the face of an outbreak; chemoprophylaxis and personal protective equipment is possible; low control measures in animal population and prevention is based on biosafety measures and culling of animals; wildlife transmission |
| **Severity of the disease in animals** |  |  | 0.6*low pathogenic avian influenza (asymptomatic or mild disease); 0.4*high pathogenic avian influenza and mortality can approach 100% within 48 hours |
| **Incidence of the disease in animals** | Hosts are present but no cases have been reported |  |  |
| **Transmission** |  |  | Transmission by direct or indirect contact; airborne transmission |

**Leptospirosis**

|  | **1** | **2** | **3** |
| --- | --- | --- | --- |
| **Severity of the disease in humans** |  | 0.9*mild influenza like with fever and chills; 0.05*meningitis, thrombocytopenia and complications; 0.05*icterohemorragiae with fatal consequences |  |
| **Economy** | Restrictions only at animal level, no hindrance to movements of animal and animal products |  |  |
| **Treatment in humans** |  |  | 0.9*effective treatment (2-6 weeks antibiotic treatment); 0.1*longer therapy with side effects (in the case of liver and kidney failure) |
| **Incidence of the disease in humans** | Sporadic incidence with 0.1-1/100,000 |  |  |
| **Control and prevention** |  | High in humans where prevention is based on washing hands and rodent control; low in animals where vaccine is available for dogs but multiple serovars possible and not cross-protective; no vaccine for cattle |  |
| **Severity of the disease in animals** | 0.8*asymptomatic with mild course of disease; 0.2*meningitis, kidney failure, chronic cases with abortion and infertility |  |  |
| **Incidence of the disease in animals** | 1-3 reported cases per year |  |  |
| **Transmission** |  | Transmission by both direct (i.e. micro-abrasions in healthy intact skin or mucous membranes) and indirect contact (water-borne outbreaks) |  |

**Listeriosis**

|  | **1** | **2** | **3** |
| --- | --- | --- | --- |
| **Severity of the disease in humans** |  | 0.4*asymptomatic or very mild course of disease; 0.4*severe illness, hospitalization is necessary and may be fatal; 0.2*fatal or severe long term damages (in risk groups or *in utero* infections) |  |
| **Economy** |  | Recall of certain products may be possible in case of outbreaks with impact on food trade |  |
| **Treatment in humans** |  |  | Long therapy (3-6 weeks antibiotic treatment); side effects are possible; relapse might occur |
| **Incidence of the disease in humans** | Sporadic with 0.6-0.8/100,000 cases; 64 cases reported in 2013 |  |  |
| **Control and prevention** |  | Moderate in humans where prevention is based on proper cleaning and sanitizing in food processing plants, HACCP programs, education of consumers and EU regulations; in animals prevention is based on attention to silage feeding |  |
| **Severity of the disease in animals** |  | 0.8*asymptomatic; 0.2*severe illness with abortion, infertility and poor prognosis |  |
| **Incidence of the disease in animals** | Sporadic incidence, around 15 reported cases/year |  |  |
| **Transmission** |  | Transmission through direct contact with infected animals and food-borne |  |

**Newcastle Disease**

|  | **1** | **2** | **3** |
| --- | --- | --- | --- |
| **Severity of the disease in humans** | Conjunctivitis and mild influenza-like symptoms |  |  |
| **Economy** | No impact |  |  |
| **Treatment in humans** | Treatment often not necessary and available if needed |  |  |
| **Incidence of the disease in humans** | Not present in Switzerland; occasional outbreaks in adjacent countries (e.g. Italy in 2012) |  |  |
| **Control and prevention** |  | High in humans where precautious can be taken to avoid direct contact; in animals prophylaxis measures and vaccines are available but it is a highly contagious disease |  |
| **Severity of the disease in animals** |  |  | 0.335*asymptomatic, very mild (lentogenic strain); 0.335*mild clinical signs but good prognosis (mesogenic strain); 0.33*fatal, herd-level mortality (velogenic strain) |
| **Incidence of the disease in animals** | Sporadic outbreaks possible in Switzerland and adjacent countries |  |  |
| **Transmission** |  |  | In humans infection is through direct inoculation of the eye; in animals rapid spread with or without animal movement; airborne transmission (described as highly contagious disease) |

**Nipah Virus Encephalitis**

|  | **1** | **2** | **3** |
| --- | --- | --- | --- |
| **Severity of the disease in humans** |  | 0.5*flu-like symptoms with fever and muscle pain; 0.25*severe illness and hospitalization is required (encephalitis with fever, coma and seizures); 0.25*fatal or severe neurological deficits |  |
| **Economy** |  |  | Restrictions at zone level: “a ban on transporting pigs within the countries affected, a temporary ban on pig production in the regions affected, as well as improvement of biosecurity practices” (OIE Manual); introduction in Switzerland would lead to a stand-still |
| **Treatment in humans** |  |  | Long therapy (supportive therapy and possible anti-virals but efficacy unknown) |
| **Incidence of the disease in humans** | Not present in Switzerland and in adjacent territories |  |  |
| **Control and prevention** |  |  | Prevention is based on early recognition of disease in animals and humans, and use of standard protective precautions |
| **Severity of the disease in animals** | Severe disease (respiratory and neurological signs) but likely not fatal |  |  |
| **Incidence of the disease in animals** | Vector and host species are not present in Switzerland or adjacent territories |  |  |
| **Transmission** |  | Direct or indirect contact (e.g. Nipah Virus contaminated date palm sap) |  |

**Q Fever**

|  | **1** | **2** | **3** |
| --- | --- | --- | --- |
| **Severity of the disease in humans** |  | 0.45*asymptomatic or very mild; 0.45*symptomatic with mild influenza signs; 0.1*complications (e.g. pneumonia, hepatitis, endocarditis and chronic infections) with abortions/neonatal deaths |  |
| **Economy** | Restrictions only at animal level |  |  |
| **Treatment in humans** |  | 0.9*effective treatment with total recovery; 0.1*long therapy |  |
| **Incidence of the disease in humans** | Sporadic infection with 0.33/100,000 cases (27 cases reported in 2013) |  |  |
| **Control and prevention** |  | In humans vaccination is possible but not easily available; in animals control is low because vaccine prevents shedding but not infection; however biosecurity measures are effective |  |
| **Severity of the disease in animals** |  | 0.7*asymptomatic and very mild course of disease; 0.15*mild clinical disease; 0.15*severe illness with increased abortion and infertility |  |
| **Incidence of the disease in animals** |  | In sheep 5% prevalence and in goats 11.1% prevalence; stable incidence rates |  |
| **Transmission** |  |  | Air-borne |

**Rabies**

|  | **1** | **2** | **3** |
| --- | --- | --- | --- |
| **Severity of the disease in humans** |  |  | Fatal |
| **Economy** |  | Restrictions at animal level (i.e. border control) and change in pet vaccination scheme |  |
| **Treatment in humans** |  |  | Post-exposure prophylaxis treatment possible immediately after exposure; the post-exposure schedule prescribes intramuscular doses of 1 ml or 0.5 ml given as four to five doses over four weeks (WHO) |
| **Incidence of the disease in humans** | Not present in Switzerland |  |  |
| **Control and prevention** | In humans prevention is available for high risk people; effective vaccination for dogs and control measures at the border |  |  |
| **Severity of the disease in animals** |  |  | Fatal |
| **Incidence of the disease in animals** | Few cases reported per year, mostly due to illegal import |  |  |
| **Transmission** | Direct contact required (aerosol transmission from bats also possible but rare and requires close contact with bats and/or bat excreta) |  |  |

**Salmonellosis**

|  | **1** | **2** | **3** |
| --- | --- | --- | --- |
| **Severity of the disease in humans** | 0.98*symptomatic and therapy is recommended but hospitalization is rare; 0.02*severe illness, hospitalization is necessary and fatal if complications arise |  |  |
| **Economy** | Restrictions on infected animals but no further restrictions on animal trade |  |  |
| **Treatment in humans** | 0.98*effective treatment and total recovery without relapse; 0.02 long therapy with limited efficacy |  |  |
| **Incidence of the disease in humans** |  |  | 15.7cases/100,000 inhabitants (1271 cases reported in 2013) |
| **Control and prevention** | Biosecurity measures are effective in preventing and controlling disease |  |  |
| **Severity of the disease in animals** | 0.8*asymptomatic; 0.2*mild clinic but good prognosis |  |  |
| **Incidence of the disease in animals** |  | Higher prevalence noted in some animal species (e.g. 15% in pigs), but low prevalence in poultry and overall rate is decreasing |  |
| **Transmission** |  | Food-borne and water-borne; direct and indirect contact |  |

**Bovine Tuberculosis (*Mycobacterium bovis*)**

|  | **1** | **2** | **3** |
| --- | --- | --- | --- |
| **Severity of the disease in humans** |  | 0.9*latent and asymptomatic; 0.1* symptomatic with severe illness; causes chronic impairment of lung function increasing susceptibility to other life-threatening diseases |  |
| **Economy** |  | Restrictions at zone level, no loss of official status |  |
| **Treatment in humans** |  |  | Long therapy |
| **Incidence of the disease in humans** | In 2013 there were 2 laboratory confirmed *M. bovis* confirmed cases |  |  |
| **Control and prevention** | Milk pasteurization; eradication scheme through testing and culling |  |  |
| **Severity of the disease in animals** |  |  | Severe illness with poor prognosis |
| **Incidence of the disease in animals** | Sporadic, but with slight increase in cases in the past years; in 2013 26 positive cattle were found on 10 farms |  |  |
| **Transmission** |  | Transmission by direct contact and food-borne |  |

**Toxoplasmosis**

|  | **1** | **2** | **3** |
| --- | --- | --- | --- |
| **Severity of the disease in humans** |  | 0.85*asymptomatic and very mild; 0.1*symptomatic; 0.05*severe illness and abortion/neonatal deaths possible in pregnant women |  |
| **Economy** | Restrictions only at animal level |  |  |
| **Treatment in humans** |  | Treatment is available for pregnant exposed women |  |
| **Incidence of the disease in humans** | Sporadic incidence (1-2 clinical cases per year in Switzerland) |  |  |
| **Control and prevention** |  | Vaccine available but it does not prevent infection; wildlife involved making control harder |  |
| **Severity of the disease in animals** | 0.9*asymptomatic in most intermediate and definitive hosts; 0.1*may cause abortion in ewes but rarely observed in Switzerland |  |  |
| **Incidence of the disease in animals** |  | Different prevalence reported in different studies; most reporting below 10% but some reporting up to 62% sero-prevalence in slaughter animals |  |
| **Transmission** |  | Transmission by direct or indirect contact |  |

**West Nile Fever**

|  | **1** | **2** | **3** |
| --- | --- | --- | --- |
| **Severity of the disease in humans** | 0.8*asymptomatic; 0.19*influenza-like symptoms with fever, headache and vomiting; 0.01*severe disease |  |  |
| **Economy** |  | Restrictions at the herd level: “the rules require that if a holding was to confirm a case of WNV, the holding is not considered free until six months have elapsed following the slaughter of the affected equidae” |  |
| **Treatment in humans** | 0.99*spontaneous recovery; 0.01*supportive therapy |  |  |
| **Incidence of the disease in humans** | Since 2010 1-2cases/year have been registered |  |  |
| **Control and prevention** |  |  | Prevention tools are difficult to implement (mosquito control) and no vaccine is available for humans; vaccine for horses available in certain countries |
| **Severity of the disease in animals** |  | 0.8*sub-clinical cases in horses; 0.1*horses develop neurological signs; 0.1*horses may die; some bird species are very susceptible with mortality rates of 20-60% |  |
| **Incidence of the disease in animals** | Disease is not present in Switzerland but has been reported in adjacent territories |  |  |
| **Transmission** |  | Vector-borne (transmission by mosquitoes and ticks) |  |
